# Supplementary material for: An ensemble deep learning model for medical image fusion with Siamese neural networks and VGG-19
Source: PLoS One. 2024 Oct 23;19(10):e0309651. doi: 10.1371/journal.pone.0309651 (PMC11498686; doi:10.1371/journal.pone.0309651)
Supplement: Attachment — Submitted filename: revision 2 comments & response.docx [file pone.0309651.s002.docx]

**Reviewers' comments:**

We would like to thank all the reviewers for their valuable comments which helps us to improve our article both technically and language-wise. Here we are addressing our responses to all the given comments.

Reviewer #1: The paper can be accepted in the current form, as authors have incorporated all the comments in the paper in proper way.

Reviewer #3:

My suggestions have not been addressed.

I ask the authors again to carefully address my points by informing me where the changes could be found in the revision.

Reviewer #3: The paper is missing of concise ordering of ideas. It is weak in describing sound research methods, statistical approaches, and interpretation. The study limitations have not been included. I would like to recommend the following points to improve the article:


**Q1**: The abstract is adequate in length and structure. The availability of the dataset used in this research effort should be highlighted in the abstract along with the salient results. The comparison with SOTA techniques should be mentioned in the abstract.

**Response:** Thank you, as per your valuable suggestion, we have modified the abstract in the revised manuscript.

**Q2**: The main innovation and contribution of this research should be clarified in the introduction. The introduction needs massive improvement to get it in an acceptable form. The motivation of the work is not clear in the introduction.

**Response:** As per the suggestions, the introduction has been modified in the revised manuscript which highlights main innovation, contribution and motivation of our research work in Section 1.

**Q3**: The proposed framework needs improvement. Figures 1 and 2 are too simple to be considered.

**Response:** Thank you for your suggestion. Figures 1 and 2 are the fundamental block diagrams, that represent the basic fusion processes with convention and deep learning-based methods.

**Q4:** Figure 3 is weakly representing the proposed method and seems in contradiction to Figures 1 and 2. The dimensional stability in the figures is also not clear due to the absence of details.

**Response:** Thank you for your valuable suggestion, the detailed information of Figure 3 is represented in Figure 6 and 7 in the revised manuscript for your kind reference.

**Q5:** Thoroughly check your article for typos and grammatical mistakes.

**Response:** Thank you, as per your valuable suggestion, we have done the language revision of our manuscript which reflects in the revised version.

**Q6:** The alignment procedure needs more text for explanation. How are tie points aligned in images of both types?

**Response:** As per your suggestion, we have incorporated the detailed image alignment process in section 3.3 in the revised manuscript.

**Q7:** Figure 4 is missing of HL and LH components of wavelet transform-based decomposition.

**Response:** Figure 4 represents the overall workflow of the proposed model, where we feel that HL and LH components are not specifically required to represent in this block diagram.

**Q8:** All the figures are of poor quality and need improvement to meet the Journal standards.

**Response:** Thank you for your valuable suggestion. All the figures quality has been improved as per the Journal standards in the revised manuscript.

**Q9:** Section 3.1 is missing data availability address details.

**Response:** As per your suggestion, data availability details are included in section 3.1 in the revised manuscript.

**Q10:** In Section 3.2, you have referred to almost all the available methods for preprocessing. Add a flowchart for these methods employed in your work with proper citations.

**Response:** Thank you, as per your valuable suggestion, we have included the flowchart of preprocessing methods along with citations (34-37) in section 3.2 as Figure 4 (b) in the revised manuscript.

**Q11:** I am not satisfied with Section 3.3. You need to show how you invoked the process.

**Response:** Thank you, as per your suggestion, The detailed image registration processes is represented in section 3.3 in the revised manuscript.

**Q12:** The lack of uniformity of format has been critically observed in the article.

**Response:** As per your suggestion, the manuscript undergone a thorough format check, and this was reflected in the revised version.

**Q13:** Section 3.4 is lacking technical details.

**Response:** As per your suggestion, the technical information of the DTCWT method highlighted in section 3.4 of the revised manuscript.

**Q14:** I am not convinced with Figure 6 which is blurred and carelessly drawn. Table 1 depicts the wrong configuration. You need to revise it.

**Response:** As per your suggestion, Figure 6 was clearly redrawn and corresponding configuration was modified in Table 1in section 3.5.1 in revised manuscript.

**Q15:** I am not satisfied with the results and analysis part of the work. It needs thorough revision.

**Response:** Thank you, as per your valuable suggestion, results section of 4.1 and 4.2 revised completely, and few additional fusion metrics were added to prove the superiority of model performance in the revised manuscript.

**Q16:** Add a subsection as “Limitations and future work recommendations” before the conclusions. Add the references in the context of future work guidelines:
<https://www.nature.com/articles/s41598-023-30309-4>
<https://doi.org/10.1016/j.inffus.2019.12.012>

**Response:** As per your valuable suggestion, “Limitations and future work recommendations” section was added before conclusions. And the given reference paper was added to feature work and citated [41] in section 7 in the revised manuscript.

**Q17:** The conclusions need thorough revision.

**Response:** Thank you, as per your valuable suggestion, the conclusion part have been modified in the revised manuscript.

Reviewer #4:

**Q1**: The author consistently emphasizes that the proposed method is founded upon a deep learning-based image fusion approach. However, the main framework of the proposed method is still based on traditional multi-scale decomposition, with the introduction of VGG-19 and SNN for feature extraction and fusion in the fusion rule setting. I believe that this method still belongs to the category of traditional multi-scale fusion methods (requiring manual parameter adjustment, not end-to-end), and it is not appropriate for the author to claim that the proposed method is based on DL.

**Response:** Thank you for highlighting the insightful key point in our work, but I strongly believe that our proposed method belongs to the deep learning model, without traditional multiscale decomposition, the proposed model will work effectively because the neural network can extract hierarchal features effectively. With the use of traditional multi scale method like wavelet transform, can provide robust muti scale feature that complement the hierarchal features learned by deep learning models. This proposed hybrid combination ensures that both local and global information that can effectively captured and fused. Majority of the contribution in proposed method is to extract complex features learned by deep learning models. Furtherly we can implement this combination for limited training data, which is computationally effective.

**Question 2:** The manuscript still lacks corresponding ablation experiments, such as the number of decomposition levels and important parameters of filters in DTCWT, as well as the effectiveness ablation experiments of VGG-19 and SNN modules.

**Response:** As per your valuable suggestion, in our proposed model, an optimal two-level decomposition is performed to capture sufficient details and minizine redundancy. It provides a moderate level of feature extraction while avoiding excessive processing and potential artifacts associated with higher levels of decomposition. Overall, the design and parameters of the filters in DTCWT play a significant role in determining the performance. In section 3.4, the detailed information related to filter size and length is presented. The ablation experiments of VGG-19 and SNN modules are presented in section 3.5.1 & 3.5.3 in the revised manuscript.

Reviewer #5: The manuscript comprehensively addresses the fusion methodology and contributing valuable insights to the field. All the suggestions are addressed by the authors.

I accept the manuscript for publication.
