# Supplementary material for: An ensemble deep learning model for medical image fusion with Siamese neural networks and VGG-19
Source: PLoS One. 2024 Oct 23;19(10):e0309651. doi: 10.1371/journal.pone.0309651 (PMC11498686; doi:10.1371/journal.pone.0309651)
Supplement: Attachment — Submitted filename: Reviewer comments & response.docx [file pone.0309651.s001.docx]

**Reviewers' comments:**

We would like to thank all the reviewers for their valuable comments which helps us to improve our article both technically and language-wise. Here we are addressing our responses to all the given comments.

Reviewer #1:

**1. Question**: Does the study present an entirely innovative methodology for integrating medical images, or does it merely amalgamate pre-existing techniques? Justify.

**Response:** Yes, the proposed methodology is an innovative approach, the fusion of medical images is done through features extracted from the hybrid deep neural network like SNN and VGG-19. As per my literature review, this methodology is not used anywhere.

**2. Question**: Have the outcomes undergone a thorough process of validation using a representative dataset and suitable metrics? Justify.

**Response:** Yes, the outcomes have undergone a thorough process of validation using a representative dataset and suitable metrics. The performance of the model is assessed using the best-suited fusion metrics, that include the Multi-scale Scheme (Q_M), Normalized Mutual Information (Q_MI), Mean Square Error (MSE), Peak Signal to Noise Ratio (PSNR), and Structural Similarity Index Measurement (SSIM), in section 4 of the revised manuscript according with suggestions.

**3. Question**: In what ways does the suggested approach enhance patient outcomes and be applicable to real-world clinical scenarios?

**Response:** In extension to this work, we proposed a tumour segmentation task, where we make of this fused image for segmentation of tumour region. It is observed that the segmentation tumour region performs well for the fused image of our proposed model with improved metrics like dice coefficient and Intersection over union compared to other resultant fused images of another model. Many guided-image surgical and treatment approaches for practical clinical settings have been enhanced through the use of fused images for assessment.

**4. Question**: Add more of the latest papers to the literature section:
<https://iopscience.iop.org/article/10.1088/1742-6596/1478/1/012024/meta>
<https://link.springer.com/article/10.1007/s13721-021-00342-2>
<https://www.taylorfrancis.com/chapters/edit/10.1201/9781003333081-4/medical-image-fusion-rajalingam-santhoshkumar-deepan-santosh-kumar-patra>
<https://www.sciencedirect.com/science/article/pii/S1746809423009679>
<https://www.sciencedirect.com/science/article/pii/S1746809421003852>

**Response:** Majority of the suggested papers are included in the literature section that helps in better improvement in literature review section 2, in the revised manuscript.

**5. Question**: Does the approach acknowledge its limitations and engage in a discussion regarding potential avenues for improvement?

**Response:** The discussion part (section 5) in the revised manuscript has been improved with potential avenues as per your suggestions.

**6. Question**: The lack of mention in the title regarding the ensemble learning implemented (e.g., stacking, boosting) may give rise to inquiries regarding the approach's originality and efficacy. More detail is required.

**Response:** Modifications have been made in section 3 of the revised manuscript as per the valuable suggestion.

Reviewer #2:

**1. Question**: Does the proposed model offer a unique approach to medical image fusion compared to existing methods? Remark it.

**Response:** Yes, the proposed ensemble model leverages the advantages of both architectures. The proposed effective extracting complementary features, hybrid learning, and potential performance improvements compared to existing fusion methods.

2. **Question**: Is the methodology clearly described and well-executed? Are the chosen architectures (Siamese networks, VGG-19) appropriate for the task? Justify.

**Response:** Yes, the methodology is clearly described in section 3 along with the proposed block diagram and workflow diagram. The executed results for various combinations are clearly mentioned in section 4 (Results section) in the revised manuscript proving that the chosen architectures Siamese networks, VGG-19 are best for image fusion tasks.

**3. Question**: Does the model achieve state-of-the-art results on relevant benchmark datasets? Are the metrics used for evaluation appropriate and informative?

**Response:** Yes, the proposed model achieves state-of-the-art results on various datasets that are publicly available. The evaluation metrics are informative. We added another 3 appropriate fusion metrics to the existing two metrics to describe the effectiveness of evaluating the model performance as discussed in section 4.1 in the revised manuscript.

**4. Question**: Literature is not up to the mark include recent literature such as Multimodality Medical Image Fusion Using Clustered Dictionary Learning in Non-Subsampled Shearlet Transform; A Non-Conventional Review on Multi-Modality-Based Medical Image Fusion; Directive clustering contrast-based multi-modality medical image fusion for smart healthcare system; Multi-modal medical image fusion in NSST domain for internet of medical things; Multimodal medical image fusion in NSST domain with structural and spectral features enhancement

**Response:** As per your suggestions, the revised manuscript incorporates most of the suggested recent papers in the literature review section 2.

**5. Question**: How well does the model perform on unseen data? Are there limitations to its generalizability?

**Response:** All the medical images in our proposed work use of two-dimensional arrays but the natural image of any random seen are generally 3-dimensional arrays having unique features for all the three (R, G, B) colour components.

**6. Question**: Is the dataset used for training and evaluation sufficiently large and diverse? Are there potential biases in the data?

**Response:** We have considered publicly available data, which is further increased by applying data augmentation like rotation, scaling, and flipping (30. 45,60).

**7. Question**: Is the model computationally efficient and feasible for real-world applications? Can the model explain its decisions and highlight the most important features in the fused image?

**Response:** Yes, model computationally efficient and feasible for real-world applications, the proposed hybrid fusion model leverages the advantages of both architectures. The proposed structure effectively extracts complementary features, allows for hybrid learning, and could improve performance. The enhanced imaging quality and robustness of the design model assist healthcare providers in enhancing medical diagnosis, treatment planning, and image-guided therapies.

**8. Question**: Does the model provide clinically meaningful insights? How would it be integrated into the clinical workflow?

**Response:** In medical image analysis such as disease diagnosis, lesion detection, and tissue segmentation can benefit from the use of this deep learning model like SNN, VGG-19, etc. By Integrating SNN with VGG-19, clinicians can enhance the performance of this task by incorporating similarity learning and pairwise comparison of medical images. The radiologist can do disease progression monitoring for better treatment by viewing the fused medical images. By Integrating SNN with VGG-19 into clinical decision support systems can assist healthcare providers in making more informed decisions.

Reviewer #3: The paper is missing of concise ordering of ideas. It is weak in describing sound research methods, statistical approaches, and interpretation. The study limitations have not been included. I would like to recommend the following points to improve the article:


1. **Question**: The abstract is adequate in length and structure. The availability of the dataset used in this research effort should be highlighted in the abstract along with the salient results. The comparison with SOTA techniques should be mentioned in the abstract.

**Response:** The suggested changes have been incorporated in the revised manuscript.

2. **Question**: The main innovation and contribution of this research should be clarified in the introduction. The introduction needs massive improvement to get it in an acceptable form. The motivation of the work is not clear in the introduction.

**Response:** The introduction part (section 1) was modified in the revised manuscript, as per the suggestions.

3. **Question**: The proposed framework needs improvement. Figures 1 and 2 are too simple to be considered.

**Response:** Figures 1 and 2 are the generalized block diagrams that show the basic difference in image fusion processes with convention and deep learning-based methods.

4. **Question**: Figure 3 is weakly representing the proposed method and seems in contradiction to Figures 1 and 2. The dimensional stability in the figures is also not clear due to the absence of details.

**Response:** The detailed information in figure 3 is represented in Figure 6 in the revised manuscript.

5. **Question**: Thoroughly check your article for typos and grammatical mistakes.

**Response:** The suggested changes have been made and reflected in the revised manuscript.

6. **Question**: The alignment procedure needs more text for explanation. How are tie points aligned in images of both types?

**Response:** The suggested changes have been made in section 3.3 in the revised manuscript.

7. **Question**: Figure 4 is missing of HL and LH components of wavelet transform-based decomposition.

**Response:** Figure 4 gives the overall work flow of the proposed model.

8. **Question**: All the figures are of poor quality and need improvement to meet the Journal standards.

**Response:** All figure quality was improved as per the Journal standards.

9. **Question**: Section 3.1 is missing data availability address details.

**Response:** Data availability details are included in section 3.1 in the revised manuscript.

10. **Question**: In Section 3.2, you have referred to almost all the available methods for preprocessing. Add a flowchart for these methods employed in your work with proper citations.

**Response:** Included flowchart of preprocessing methods along with citations (34-37) in section 3.2 in the revised manuscript.

11. **Question**: I am not satisfied with Section 3.3. You need to show how you invoked the process.

**Response:** The suggested changes have been made in section 3.3 in the revised manuscript.

12. **Question**: The lack of uniformity of format has been critically observed in the article.

**Response:** The suggested changes have been made and reflected in the revised manuscript.

13. **Question**: Section 3.4 is lacking technical details.

**Response:** The suggested changes have been made in section 3.4 of the revised manuscript

14. **Question**: I am not convinced with Figure 6 which is blurred and carelessly drawn. Table 1 depicts the wrong configuration. You need to revise it.

**Response:** The suggested changes have been made in section 3.5.1 in the revised manuscript.

15. **Question**: I am not satisfied with the results and analysis part of the work. It needs thorough revision.

**Response:** The results section was revised, and a few additional fusion metrics were added in the revised manuscript for better analysis as per the suggestion.

16. **Question**: Add a subsection as “Limitations and future work recommendations” before the conclusions. Add the references in the context of future work guidelines:
<https://www.nature.com/articles/s41598-023-30309-4>
<https://doi.org/10.1016/j.inffus.2019.12.012>

**Response:** Changes have been made in the revised manuscript in section 7.

17. **Question**: The conclusions need thorough revision.

**Response:** The conclusion part has been modified in the revised manuscript as per the suggestion.

Reviewer #4: The author proposes an ensemble learning-based image fusion model using a Siamese neural network (SNN) and VGG-19 and achieves specific results in PSNR and SSIM metrics.

1. **Question**: The figures presented in the paper are unclear; therefore, it is recommended that vector graphics with a resolution of 600 dpi be used to enhance their clarity and quality.

**Response:** Modifications are done as per the suggestions in the revised manuscript.

2. **Question**: The paper's contribution is relatively weak, lacking in innovation, and numerous similar methods were developed several years ago.

**Response:** Even though various similar methods are developed on traditional multi-scale decomposition methods, our work proposes an efficient hybrid deep learning model for medical image fusion using pretrained and non-pretrained networks i.e. VGG-19 and SNN with stacking ensemble method. His hybrid model has better performance than existing works as discussed in results section 4.

3. **Question**: The methods used for comparison are all traditional multi-scale decomposition methods, lacking comparison with related deep learning-based methods.

**Response:** we added CNN and vgg-19 deep learning-based methods along with traditional methods in section 4 as per suggestions.

4. **Question**: The paper only compares based on the objective evaluation metrics of PSNR and SSIM, which cannot demonstrate the effectiveness of the proposed method. Please add corresponding evaluation metrics.

**Response:** To effectively evaluate the proposed model performance the most important fusion metrics like Mean Square Error (MSE), Normalized Mutual Information (Q_MI), and Multi-scale Scheme (Q_M) are included along with PSNR and SSIM as per the suggestions in section 4.

5. **Question**: The experimental section of the author's work is deficient in relevant ablation experiments, such as exploring various fusion rules for the proposed method. This would enhance the rigor and comprehensiveness of the study.

**Response:** To bias, the equal contribution of two paraments are considered during the fusion processes. This approach avoids the need for a complex algorithm to determine the relative importance of each input image, which leads to computational efficiency. By treating all the input images equally, the image fusion processes is less sensitive to variation in image characteristics, contrast, brightness, and Nosie levels.

Reviewer #5: The article is suitable for publication, after the author addresses and incorporates the following in the manuscript


1. **Question**: How are the components of the medical images decomposed using the Dual-Tree Complex Wavelet Transformation (DTCWT)-based multi-scale image decomposition?

**Response:** The detailed image decomposition using DTCWT) is included in section 3.5 in the revised manuscript.

2. **Question**: How are these fusion rules determined, and what criteria are used to select the most appropriate rule for each component?

**Response:** Fusion rules in the context of image fusion are determined based on the specific needs and characteristics of the system or application. fusion rules are selected based on the following considerations. The fusion rule should be robust to variations and uncertainties in the input data, as well as changes in the environment or operating conditions. The computational complexity of the fusion rule should be manageable, especially in real time. In some cases, there may be criteria for optimality, such as minimizing estimation error or maximizing information gain. There is a trade-off between these criteria and the most appropriate rule may vary depending on the context and constraints of the problem.

3. **Question**: How does the model perform active level measurement and fusion rules jointly in a single step?

**Response:** Detailed information regarding active level measurement and fusion rules jointly in a single step is present in the introduction part section 1. In the revised manuscript.

4. **Question**: How does the model generalize to unseen datasets or different combinations of source images?

**Response:** The fusion of SNN and VGG-19 (CNN with 19 deep layers) involves combining different learning mechanisms. CNNs typically use backpropagation for training, while SNNs can be trained using methods like Spike-Timing-Dependent Plasticity (STDP). Combining these learning mechanisms can potentially lead to better generalization and adaptation to different types of data

5. **Question**: Can you explain in more detail how the weight map is generated for the base information in the fusion process?

**Response:** The detailed explanation is given in section 3.5.2 in the revised manuscript.

6. **Question**: What factors or characteristics of the source images are considered in determining the weight values for each pixel?

**Response:** The weight values of each pixel are determined based on several factors of the source images like Pixel Intensity, Texture, Contrast and Edge Information. The intensity of each pixel in the source images can influence its weight value in the fused image. Pixels with higher intensity may be given more weight and vice versa. Texture features, such as local variations in pixel intensity, can indicate the importance of certain regions in the source images. The contrast between neighbouring pixels within each source image can affect their weight values. Regions with higher contrast may be considered more significant for fusion. Pixels along edges may be assigned higher weight values to preserve important details in the fused image. These factors are often incorporated into fusion algorithms to dynamically adjust the weight values of each pixel in the fused image, ensuring that important information from all source images is appropriately represented.

7. **Question**: Are there any specific challenges or complexities involved in calculating the weight map, especially across multiple source images with different modalities?

**Response:** The following challenges are involved in calculating the weight map when dealing with multiple image modality fusion. Aligning images from different modalities can be challenging due to differences in spatial resolution, orientation, and other factors. While it can be difficult, establishing relationships between features taken from multiple modalities is crucial for an effective weight map calculation. The final weight map's decision regarding how much weight to give each modality is subjective and can call for empirical testing.

8. **Question**: Are there any considerations or adjustments made to account for differences in saliency between different types of medical images?

**Response:** The model parameters are fine-tuned for the architectures. The base visual features like edges, corners, and textures are extracted using the SNN model and the detailed features like complex patterns, and shapes are extracted using deep learning of the VGG-19 model. Anisotropy diffusion filters are employed to improve the edges of image modality. In addition, pre-processing techniques like noise reduction, and contrast enhancement are considered to increase the saliency between different types of medical images.

9. **Question**: Can you explain the significance of the weighted sum pixel calculation equation in the fusion process?

**Response:** Explained the significance of the weighted sum pixel calculation equation in the fusion process in section 3.5.2 in the revised manuscript.

10. **Question**: How do the weighting factors (α1 and α2) affect the contribution of each source image to the fused base part?

**Response:** The importance of selecting the weighting factors is discussed in section 3.5.2 In the revised manuscript.

11. **Question**: Are there any considerations or trade-offs involved in selecting the values of α1 and α2, such as preserving common features and reducing redundant information?

**Response:** The weighting factor value ranges from 0 to 1. For uniformity in fusion as well to preserving common features and reducing redundant information the trade-off involved in selecting the values of α1 and α2 is 0.5.

12. **Question**: Can you provide insights into how the fused base part captures and integrates information from both source images while preserving common features?

**Response:** In the fusion process, the fused base part captures and integrates information from both source images by utilizing features extracted from each source image. Here each source image is passed through a two-parallel Convolutional Neural Network (CNN) network, for feature extraction which identifies and extracts relevant features from the input images. The extracted features are fused using a weighted sum fusion strategy to create a unified representation that preserves common features. This fusion step ensures that important information from each image contributes to the final output while minimizing redundancy and preserving common features. The amount of information contribution from the source image depends on the weighting factor. For uniform contribution here we consider α is equal to 0.5.

13. **Question**: What techniques or approaches are used to optimize the parameters of the Siamese network and pre-trained CNN model for weight map generation?

**Response:** SoftMax operation is used to calculate the probability of error between the parallel network in SNN and the gradient descent algorithm is used to optimize the parameters of the Siamese network and pre-trained CNN model for weight map generation

14 **Question**: Required a detailed ablation study.

**Response: An** ablation study for weight map calculation is added in sections 3.5.2 and 3.5.2 in the revised manuscript.

6. PLOS authors have the option to publish the peer review history of their article ([what does this mean?](https://journals.plos.org/plosone/s/editorial-and-peer-review-process#loc-peer-review-history)). If published, this will include your full peer review and any attached files.


If you choose “no”, your identity will remain anonymous, but your review may still be made public.


**Do you want your identity to be public for this peer review?** For information about this choice, including consent withdrawal, please see our [Privacy Policy](https://www.plos.org/privacy-policy).

Reviewer #1: No

Reviewer #2: No

Reviewer #3: **Yes:** Prof Dr Shahzad Ahmad Qureshi

Reviewer #4: No

Reviewer #5: **Yes:** Dr. Laavanya Mohan
